# On the Origin of the Term Man-Midwife

**Published:** 1811-07

**Authors:** 


*To the Editors of the Medical and Physical Journal.*

### *On the Origin of the Term Man-midwife.*

Gentlemen,

I SHOULD be much obliged to any of your Correspondents for information when the compound word *Man-midwife* was first used, and by whom?

It is inserted in Ainsworth's dictionary, 1736, immediately below the word *Midwife*, and is explained, *Medicus parturientibus opem ferens*. It is likewise inserted in Boyer's French dictionary, 1742, but it is not in Johnson's English dictionary.

Sir Fielding Ould, in 1742, writes himself *Man-midwife*; Smellie has a chapter on the Qualifications of an *Accoucheur*, thus rejecting the term *Man-midwife*.

In 1705 was published "Portal's complete Practice of *Men* and *Women Midwifes*."

In "The compleat Midwife's Practice," published in 1656, this expression is not to be found, though the ladies are censured "for making election of men to bring them to bed," which is said to be "a great piece of impudence, unless it be in a case of very great danger:" the word *chirurgeon* is throughout this book used to designate the *accoucheur*.

Dr. Hugh Chamberlain, who translated "Mauriceau's Diseases

Diseases of Women with Child and in Child-bed," 1672, employs the term "*Artist in Midwifery.*"

From this it may be supposed that the word Man-midwife began to be used between 1672 and 1705; but for the express time I must refer to some other correspondent.

OBSTETRICUS.

*May* 10, 1811.

